# Effect of Artificial Neural Network Design Parameters for Prediction of PS/TiO_2_ Nanofiber Diameter

**DOI:** 10.3390/polym18030328

**Published:** 2026-01-26

**Authors:** R. Seda Tığlı Aydın, Fevziye Eğilmez, Ceren Kaya

**Affiliations:** Department of Biomedical Engineering, Zonguldak Bülent Ecevit University, Incivez, Zonguldak 67100, Turkey

**Keywords:** TiO_2_, polystyrene, electrospinning, nanofiber, prediction, ANN

## Abstract

In this study, polystyrene (PS) and PS/TiO_2_ nanofibers were fabricated through electrospinning and quantitatively characterized to analyze and predict fiber diameters. To advance predictive methodologies for materials design, artificial neural network (ANN) models based on multilayer perceptron (MLP) and radial basis function (RBF) architectures were developed using system- and process-level parameters as inputs and the fiber diameter as the output. Two data classes were constructed: Class 1, consisting of PS/TiO_2_ nanofibers, and Class 2, containing both PS and PS/TiO_2_ nanofibers. The architectural optimization of the ANN models, particularly the number of neurons in hidden layers, had a critical influence on the correlation between predicted and experimentally measured fiber diameters. The optimal MLP configuration employed 40 and 20 neurons in the hidden layers, achieving mean square errors (MSEs) of 4.03 × 10^−3^ (Class 1) and 7.01 × 10^−3^ (Class 2). The RBF model reached its highest accuracy with 30 and 250 neurons, yielding substantially lower MSE values of 1.42 × 10^−32^ and 2.75 × 10^−32^ for Class 1 and Class 2, respectively. These findings underline the importance of methodological rigor in data-driven modeling and demonstrate that carefully optimized ANN frameworks can serve as powerful tools for predicting structural features in nanostructured materials, thereby supporting rational materials design and synthesis.

## 1. Introduction

Preparation and characterization of nanomaterials such as nanofibers with a tailorable size, shape, and surface chemistry play an important role in diverse applications, including biomedical engineering [1,2] as well as electrical, optical, and energy-related technologies [3,4,5]. Precise control over nanomaterial dimensions and morphology is essential for tailoring properties to meet application-specific requirements. For instance, the dimensions and morphological structure of the electrospun nanofibers, fabricated via the electrospinning process, are of great interest as they influence properties such as flexibility, wettability, and biological performance, making them promising candidates for applications in tissue engineering and drug delivery [6,7]. Electrospinning, a widely used technique for nanofiber fabrication, is highly sensitive to processing parameters such as applied voltage, flow rate, and nozzle-to-collector distance. Optimizing these parameters is crucial for achieving uniform fiber diameters and well-defined morphologies [8]. However, the experimental investigation required to optimize the process parameters involves many tests and iterations, typically relying on extensive trial-and-error studies. Although certain trends seem to be well-known, such as the relationships between applied voltage and resulting nanofiber diameter, these experiments are time consuming and expensive [9]. Furthermore, the complex interplay of the electrospinning method, polymer properties, and manipulation of the environmental conditions simultaneously affects the nanofiber diameter [10,11]. Consequently, efficient predictive approaches are required to guide nanofiber fabrication by identifying optimal processing conditions or predicting fiber dimensions.

Artificial neural networks (ANNs) have emerged as powerful tools for modeling nonlinear systems since they can substantially reduce time, labor, and material costs by predicting the nanofiber size from processing parameters [12,13,14,15]. Thus, the necessity for the prediction of nanofiber size has accelerated ANN-based modeling studies, and researchers have paid great attention to ANN models due to their successful predictions in this area [10,16,17,18,19,20,21,22,23,24,25]. Sarkar et al. investigate neural network model-based feedback control techniques to regulate the polyethylene oxide (PEO) nanofiber diameter in an electrospinning process from published experimental data [16]. Khanlou et al. used an ANN model for the prediction of polymethyl methacrylate (PMMA) nanofibers with the parameters of the studied polymer concentration, feed rate, and nozzle–collector distance [17]. Nasouri et al. trained three parameters (polymer concentration, applied voltage, and nozzle–collector distance) for the prediction of polyacrylonitrile (PAN) electrospun nanofibers, and showed that the predicted values differed by only 4.4% from experimental results [18]. Recently, Ma et al. employed ANN models, enabling precise predictions of PVDF fiber membrane thickness under varying electrostatically spun conditions [26]. Furthermore, several studies have investigated the prediction of the diameters of several nanofibers: nylon-6,6 [19], chitosan/polyvinyl alcohol (PVA) [20], polyurethane (PU) [21], chitosan/polyethylene oxide (PEO) [22], polycaprolactone (PCL) [23], polyvinylpyrrolidone (PVP) [24], and PCL/gelatin nanofibers [10,25].

Several studies noted that electrospun polymer nanofiber membranes are widely used in filtration, antibacterial coatings, and self-cleaning surfaces due to their high surface area, interconnected porosity, and tunable morphology [27,28,29,30,31]. Moreover, titanium dioxide (TiO_2_) has gained attention as a nanofiber additive due to its photocatalytic activity, biocompatibility, antimicrobial properties, and low cost [32,33]. Polystyrene (PS) combined with titanium dioxide (TiO_2_) forms multifunctional nanofibers that couple mechanical stability with photocatalytic and antimicrobial activity, making PS/TiO_2_ membranes attractive for air and water filtration and antibacterial surfaces. PS/TiO_2_ nanofiber membranes also have direct applications in the adsorption of heavy metal ions from water, demonstrating their potential for environmental remediation [34], or can additionally be utilized as functional fillers for the reinforcement of a polymer matrix [35]. Previously, PS/TiO_2_ nanofibers have been investigated in terms of fiber morphology and thermal properties [35,36], as well as antibacterial properties [37]. In such systems, the fiber diameter also controls the exposure of TiO_2_ nanoparticles, directly affecting the functional performance. However, the fiber diameter is highly sensitive to electrospinning parameters, and its optimization by trial and error is inefficient. Madani et al. emphasized the challenges associated with the formation of PS/TiO_2_ nanofibers, noting that even minor variations in the electrospinning solution composition can lead to significant changes in the final fiber morphology [36]. Consequently, purely experimental optimization of PS/TiO_2_ nanofiber membrane fabrication is inefficient in the absence of a more systematic process optimization strategy. Therefore, data-driven predictive models are needed to enable rational design of PS/TiO_2_ electrospun membranes with targeted properties. Although several nanofibers have been studied thus far, to the best of our knowledge, the prediction of PS and PS/TiO_2_ electrospun nanofibers’ diameters using ANN algorithms remains unexplored, representing a clear gap addressed by this study.

A key challenge in ANN applications is the lack of standardization in network architectural design [23,38]. Previous studies reported that the prediction of experimental data can be favored by the use of a single hidden layer [39]; however, more complex structures involving multiple hidden layers or varying neuron numbers often achieve superior accuracy [38,40,41]. Nevertheless, the determination of the optimal network architecture typically relies on empirical, trial-and-error approaches, which are computationally expensive [42]. Thus, according to the research hypothesis, the investigation of ANN model architectural design based on producing the best results will enable the successful prediction of PS/TiO_2_ nanofiber size without the need for further trials during ANN structural design, which is the main obstacle related to the computational requirements for learning.

In this study, we fabricated PS and PS/TiO_2_ nanofibers using the electrospinning method under various operational process parameters (applied voltage, flow rate, distance between the nozzle and collector, and solvent type), and the prepared nanofibers were imaged in order to measure the nanofiber diameter. The resulting nanofibers were characterized by FTIR and XRD, and activity was tested against Gram-negative Escherichia coli (*E. coli*) and Gram-positive Staphylococcus aureus (*S. aureus*) bacteria. Then, we introduced the outputs of measured nanofiber diameters as outputs for two ANN models (MLP: multilayer perceptron and RBF: radial basis function) to predict nanofiber diameters of PS/TiO_2_ and the total PS and PS/TiO_2_. Model performance was evaluated using the mean square error (MSE), mean absolute error (MAE), root mean square error (RMSE), correlation coefficient (R), and coefficient of determination (R^2^). The novelty of this work lies in the first application of ANN-based predictive modeling to PS and PS/TiO_2_ electrospun nanofibers, coupled with a systematic comparison of MLP and RBF architectures and the development of practical guidelines for optimizing ANN design to improve the prediction accuracy in nanomaterial fabrication. The results not only provide the first ANN-based prediction of PS/TiO_2_ nanofiber diameters but also establish guidelines for ANN architectural design by varying hidden neurons and spread parameters to achieve the optimal predictive accuracy.

## 2. Materials and Methods

Polystyrene (PS, M_W_:192.000) was obtained from Sigma-Aldrich Chemical Co., Ltd. (Hamburg, Germany). TiO_2_ (titanium (IV) oxide, anatase, nanopowder, <25 nm particle size, 99.7% trace metals basis, Catalogue no. 637254), N,N,-dimethylformamide (DMF), tetrahydrofuran (THF), and acetone were of analytical grade and purchased from Sigma-Aldrich Chemical Co. Ltd. (Hamburg, Germany).

### 2.1. Preparation of PS and PS/TiO_2_ Nanofibers

Homogeneous polymer solutions were prepared by dissolving 10% (*w*/*v*) PS in two different solvent mixtures of DMF:THF (60:40, *v*/*v*) and DMF:acetone (80:20, *v*/*v*). PS/TiO_2_ solutions were prepared by dissolving 10% (*w*/*v*) PS and 5% (*w*/*v*) TiO_2_ in solvent mixtures of DMF:THF (60:40, *v*/*v*). Then, they were stirred overnight at room temperature to obtain an electrospinning solution. The prepared PS solutions with two different solvent mixtures were loaded into a 10 mL syringe supplied with a blunt stainless-steel 21G needle (inner diameter of 0.8 mm), then mounted on a syringe pump (New Era Pump Systems, Model No: NE-300) to control the polymer flow rate. A high DC voltage in the range of 19–24 kV was applied between the needle and the grounded collector to initiate the electrospinning jet, and PS nanofibers were collected under ambient temperature and humidity conditions. Three processing parameters (applied voltage, solution flow rate, and needle-to-collector distance) were experimentally optimized to obtain uniformly distributed nanofibers without bead formation on the membranes. The polymer solution flow rate was controlled using a syringe pump and adjusted between 0.05 and 0.1 mL/min, while the needle-to-collector distance was varied between 10 and 15 cm. Processing parameters were optimized with regard to homogenously distributed nanofibers without any beading formation. The selection of the processing parameter ranges was guided by previously reported studies [27,28,43,44].

### 2.2. Imaging PS and PS/TiO_2_ Nanofibers and Nanofiber Diameter Size Analysis

The morphology and surface topography of PS and PS/TiO_2_ nanofiber membranes were examined using a scanning electron microscope (SEM, QUANTA 450, Thermo Fisher Scientific, OR, USA) after sputter-coating with a thin gold–palladium layer under vacuum. Fiber diameters (*n* ≈ 30) were determined from SEM images using JMicroVision 1.2.7 (National Institutes of Health, Bethesda, MD, USA).

### 2.3. Characterization and Antibacterial Activity of PS and PS/TiO_2_ Nanofibers

PS and PS/TiO_2_ nanofibers with the smallest fiber diameters were selected because a reduced fiber diameter increases the specific surface area and exposure of TiO_2_ nanoparticles, which are critical for structural characterization and antimicrobial activity. Fourier transform infrared (FTIR) spectra were recorded using a spectrophotometer (Bruker IFS 66/S, Germany) in the range of 500–4000 cm^−1^ to confirm the incorporation of TiO_2_ within the nanofiber membranes. X-ray diffraction (XRD) patterns were obtained using a PANalytical Empyrean diffractometer over a 2θ range of 10–90°, with a step size of 0.026° and a dwell time of 93.84 s per step. The antimicrobial activity of the nanofibers was evaluated against Staphylococcus aureus (ATCC 6538, Gram-positive) and *Escherichia coli* (ATCC 25922, Gram-negative) using the zone of inhibition method [1]. Bacterial strains were cultured in Tryptic Soy Broth overnight at 37 °C under aerobic conditions, and the final inoculum was adjusted to approximately 10^5^ CFU/mL. Tryptic Soy Agar plates were prepared, and membranes (∼6 mm in diameter) were UV-sterilized for 30 min before being placed at the center of the inoculated agar plates. Plates were incubated at 37 °C (pH = 7.4) for 24 h, and then inhibition zones (D, mm) were measured. All assays were performed in triplicate.

### 2.4. Artificial Neural Network (ANN) Modeling

**Figure 1:**
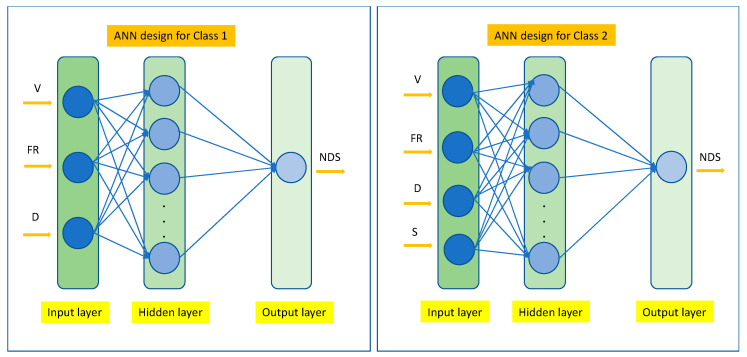
ANN design architecture for prediction of nanofibers representing Class 1 (PS/TiO_2_ nanofibers) and Class 2 (PS and PS/TiO_2_ nanofibers). Voltage: V, flow rate: FR, distance between the needle tip and the collector: D, solvent type: S, mean nanofiber diameter size: NDS.

The ANN is a computational model composed of interconnected nodes designed to address complex problems involving nonlinear relationships between input variables and output responses [45]. A typical ANN architecture comprises three layers: an input layer representing independent variables, an output layer corresponding to dependent responses, and one or more hidden layers that link inputs to outputs [13]. In this study, 2 classes of input layers (Class1: PS/TiO_2_ nanofibers, Class 2: PS and PS/TiO_2_ nanofibers) were evaluated for the responses of nanofiber diameter size. In the first class, the input layer included 3 independent variables, which were the voltage (V), flow rate (FR), and distance between the needle tip and the collector (D). In the second class, PS nanofibers fabricated with DMF:THF solvent and DMF:acetone solvent, and PS/TiO_2_ nanofibers fabricated with DMF:THF solvent were evaluated for 4 input layer layers: the voltage (V), flow rate (FR), distance between the needle tip and the collector (D), and solvent type (S). The mean nanofiber diameter size (NDS) was defined as the output variable. Class 2 was constructed by combining PS nanofibers prepared in DMF:THF and DMF:acetone with PS/TiO_2_ nanofibers prepared in DMF:THF in order to train the ANN on both solvent-driven and TiO_2_-driven variations in electrospinning behavior. By merging these datasets, the ANN is trained on a broader and more physically representative parameter space rather than correlations specific to a single material or solvent system. For both classes, a single hidden layer with a designated number of neurons was employed. A fully connected network architecture was constructed to allow information transfer between neurons. The schematic representation of the ANN model developed for nanofiber diameter prediction was generated using the nntool command in MATLAB R2016a (Figure 1). To enhance model reliability, k-fold cross-validation was applied in combination with the hold-out method.

#### 2.4.1. Datasets and Data Collection

Training and test datasets were constructed from nanofiber attributes measured using ImageJ (NIH image program, version 1.49) software. The dataset was randomly divided into 80% for training and 20% for testing. For Class 1 (PS/TiO_2_ nanofibers), 32 data were used. For Class 2, a total of 267 data were available: 99 from PS nanofibers prepared with DMF:THF solvent, 136 from PS nanofibers prepared with DMF:acetone solvent, and 32 from PS/TiO_2_ nanofibers fabricated with DMF:THF solvent. The ANN architectures and training designs are summarized in Table 1.

#### 2.4.2. Multilayer Perceptron (MLP) Network Model, Design Factors, Training Parameters

The multilayer perceptron (MLP) is a feed-forward neural network comprising an input layer, one or more hidden layers, and an output layer. Each layer contains processing units (neurons) fully interconnected through weighted connections to subsequent layers. During training, the back-propagation algorithm iteratively updates connection weights to minimize the difference between predicted and target outputs [17,46]. For a neuron in the output layer, the output $x_{0}$ is expressed as [38,47]

(1) $$x_{0}=f\left(\sum_{n}x_{n}\omega_{no}\right)$$

where *f*() is the activation function, $x_{n}$ is the activation of the *n*th hidden layer node, and $\omega_{no}$ is the interconnection between the *n*th hidden layer node and the *o*th output layer node. The most common activation function in the back-propagation algorithm is the sigmoid functions, expressed as [38,47]

(2) $$x_{0}=\frac{1}{1+\exp\left(-\sum x_{n}\omega_{no}\right)}$$

In this study, MLP models were developed for all nanofiber classes, with the nanofiber diameter size as the output. Input data were normalized to the [0, 1] range. The MLP design parameters included the number of neurons, activation function, training function, and number of iterations. The training function was set to Levenberg–Marquardt, and the activation function was chosen as tangent sigmoid (tansig) for both hidden and output layers. The effect of the neuron number (2–40) was investigated under a maximum of 1000 epochs (Table 1).

#### 2.4.3. Radial Basis Function (RBF) Network Model, Design Factors, Training Parameters

RBF neural networks, which share a similar structure to MLP models, offer fast learning, high accuracy, and a relatively simple topology. Unlike MLPs, the hidden nodes employ radially symmetric basis functions as activation functions. The spread constant, defining the center and width of the Gaussian function, is given by [38,48]

(3) $$\Phi_{j}(x)=\exp\left(-\frac{{∥x-\mu_{j}∥}^{2}}{{2\sigma_{j}}^{2}}\right)$$

where $\Phi_{j}(x)$ is the nonlinear function of unit j, $\mu_{j}$ is RBF function center and x the input data vector, and $\sigma_{j}$ is the spread of the Gaussian basis function. The spread constant is determined empirically through trial and error within the network architecture. An RBF neuron attains its maximum output when the input vector coincides with its center, and the overall network performance is evaluated after the training process [10,38,47].

In this study, an RBF neural network model was developed for all nanofiber classes to maximize prediction accuracy. The architecture was designed by tuning key training parameters, namely the number of neurons and the spread constant. Model performance was evaluated by varying the number of neurons during training while fixing the spread constant at 0.1 (Table 1).

#### 2.4.4. Model Evaluation

The predictive performance of MLP and RBF models was assessed using statistical indicators, namely mean squared error (MSE), root mean squared error (RMSE), mean absolute error (MAE), and the coefficient of determination (R^2^), calculated according to Equations (4)–(7) [10,38] presented below:

(4) $$MSE=\frac{\sum_{i=1}^{n}{\left(yi-\hat{y}i\right)}^{2}}{n}$$

(5) $$RMSE=\sqrt{\frac{\sum_{i=1}^{n}{\left(yi-\hat{y}i\right)}^{2}}{n}}$$

(6) $$MAE=\frac{\sum_{i=1}^{n}\left|yi-\hat{y}i\right|}{n}$$

(7) $$R^{2}=\frac{\sum_{i=1}^{n}{\left(\hat{y}i-\bar{y}i\right)}^{2}}{\sum_{i=1}^{n}{\left(yi-\bar{y}i\right)}^{2}}$$

where yi and $\hat{y}i$ are the target (observed) and network output (predicted) values of the nanofiber diameter size, $\bar{y}i$ is the mean of the target values, and n represents the number of experiments.

## 3. Results

### 3.1. Morphology and Diameter of PS and PS/TiO_2_ Nanofibers

**Figure 2:**
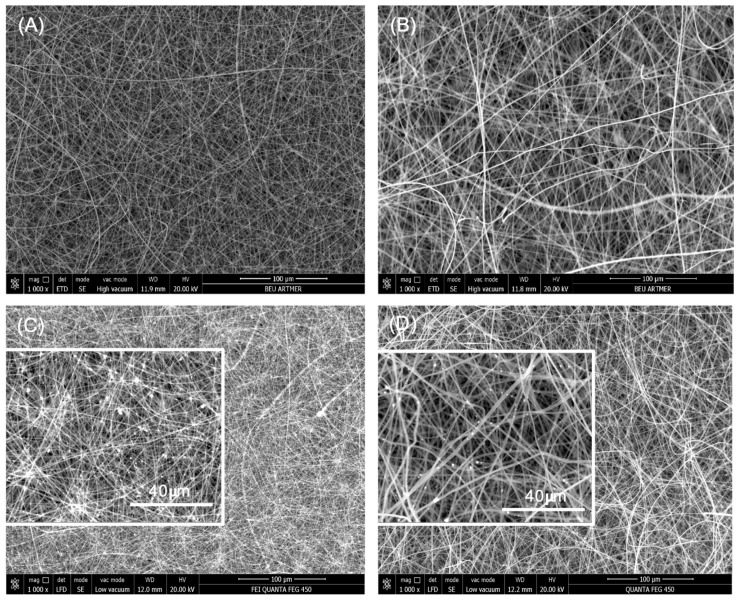
SEM images of electrospun PS and PS/TiO_2_ nanofibers fabricated under different solvent systems and electrospinning parameters: (**A**) PS nanofibers prepared using DMF/THF solvent (0.07 mL/min, 23 kV, 12 cm) (1000×); (**B**) PS nanofibers prepared using DMF/acetone solvent (0.10 mL/min, 20 kV, 15 cm) (1000×); (**C**) PS/TiO_2_ nanofibers prepared using DMF/THF solvent (0.05 mL/min, 19 kV, 10 cm) (1000× and 4000×); and (**D**) PS/TiO_2_ nanofibers prepared using DMF/THF solvent (0.10 mL/min, 23 kV, 15 cm) (1000× and 4000×).

The preparation of PS and PS/TiO_2_ nanofibers is based on an electrospinning technique for the production of polymer fibers at a micro- or nanoscale [49]. During the electrospinning process, nanofiber uniformity (without bead formation) and size are greatly influenced by the system parameters (polymer concentration, viscosity, surface tension and conductivity) and process parameters (electric voltage, flow rate and distance between the capillary and collector) [9,50,51]. In this study, PS nanofibers were successfully prepared using two different solvent mixtures, DMF:THF and DMF:acetone, under varying process parameters. A total of 235 data on uniform PS nanofibers were maintained, which were available for particle imaging. Figure 2 shows SEM images of PS nanofibers with the smallest fiber diameter (356.9 ± 25 nm) (Figure 2A) and the largest fiber diameter (1196.1 ± 30 nm) (Figure 2B) fabricated under the designated process parameters.

PS/TiO_2_ nanofibers were fabricated via the same method as in PS nanofibers keeping the DMF:THF solvent type, chosen with regard to the smallest nanofiber diameter of PS nanofibers with varying process parameters. Thus, a total of 32 data on PS/TiO_2_ nanofibers were maintained, which were available for particle imaging without any beading formation. Figure 2C,D demonstrate SEM images of electrospun PS/TiO_2_ nanofibers with the smallest nanofiber diameter of PS/TiO_2_ nanofibers (283.35 ± 30 nm) (Figure 2C) and the largest nanofiber diameter of PS/TiO_2_ nanofibers (716.8 ± 45 nm) (Figure 2D) under varying process parameters.

### 3.2. Structural Characterization and Antibacterial Activity of PS and PS/TiO_2_ Nanofibers

**Figure 3:**
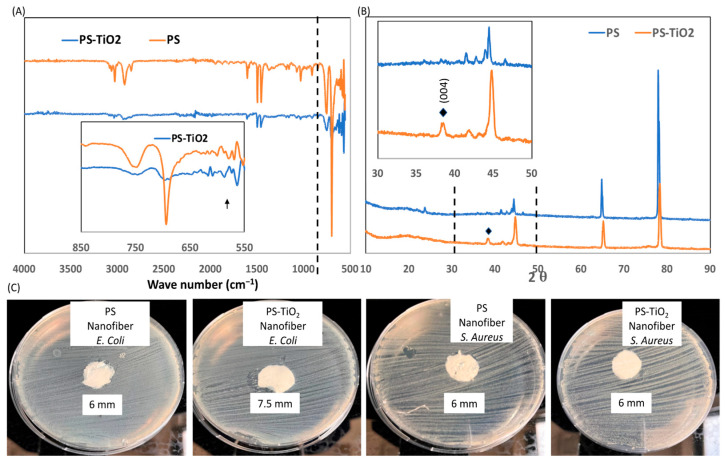
(**A**) FTIR spectra and (**B**) XRD diffraction patterns of PS and PS/TiO_2_ nanofibers, (**C**) zone inhibition assay images of *S. aureus* and *E. coli* in the presence of PS and PS/TiO_2_ nanofibers. The blank disk diameter is 6 mm.

Structural characterization of PS and PS/TiO_2_ nanofibers was evaluated by FTIR spectra of PS and PS/TiO_2_ nanofibers, as presented in Figure 3A. Neat PS exhibited characteristic absorption bands corresponding to aromatic C–H stretching vibrations at ~3025 cm^−1^, aliphatic C–H stretching at 2850–2920 cm^−1^, and C=C stretching of the phenyl ring at ~1600 cm^−1^, confirming the preservation of the polymer backbone during electrospinning [52]. The FTIR spectra (Figure 3A) revealed a characteristic absorption band at ~580 cm^−1^ in PS/TiO_2_ nanofibers, corresponding to Ti–O stretching vibrations, which indicates the presence of TiO_2_ nanoparticles within the PS matrix [53].

The crystalline structure of the embedded TiO_2_ nanoparticles was further investigated by XRD analysis (Figure 3B). A diffraction peak at 2θ ≈ 38° was observed for PS/TiO_2_ nanofibers, consistent with the anatase phase of TiO_2_ (JCPDS 21–1272) [54]. This confirms that the TiO_2_ nanoparticles retained their anatase crystalline phase after electrospinning. Incorporating TiO_2_ nanoparticles into the PS matrix has been shown to cause slight shifts in XRD peak positions, which may due to interfacial interactions and changes in lattice structure in the composite system, as observed in other TiO_2_–polymer nanocomposites [31,55]. Such structural retention of anatase TiO_2_ is favorable for the intended functional performance of the nanofiber membranes.

The antibacterial performance of PS and PS/TiO_2_ composites was evaluated using a zone inhibition assay against *S. aureus* and *E. coli* (Figure 3C). No inhibition zones were detected for either PS or PS/TiO_2_ nanofibers against S. aureus, indicating no measurable antibacterial activity toward this Gram-positive strain under the tested conditions. In contrast, a small inhibition zone was observed for E. coli in the presence of PS/TiO_2_ nanofibers, with the zone diameter increasing from the blank disc value of 6 mm to approximately 7.5 mm. This modest increase is consistent with the known antibacterial tendency of TiO_2_ toward Gram-negative bacteria such as *E. coli* [56], but the magnitude of the effect is limited. Therefore, the results indicate a preliminary antibacterial response rather than a strong bactericidal effect, suggesting that PS/TiO_2_ nanofibers may provide supplementary antimicrobial functionality.

### 3.3. MLP Network Modeling

In the MLP learning algorithm, the connection weights of neurons in the hidden layers are continuously updated, with each layer consisting of multiple processing units. In this study, the effect of varying the number of neurons in the hidden layer was examined by comparing the differences between target and network output samples for two classes of nanofibers, with the aim of achieving the highest prediction accuracy.

#### 3.3.1. Prediction Performance of Class 1 and Class 2 Nanofibers

**Figure 4:**
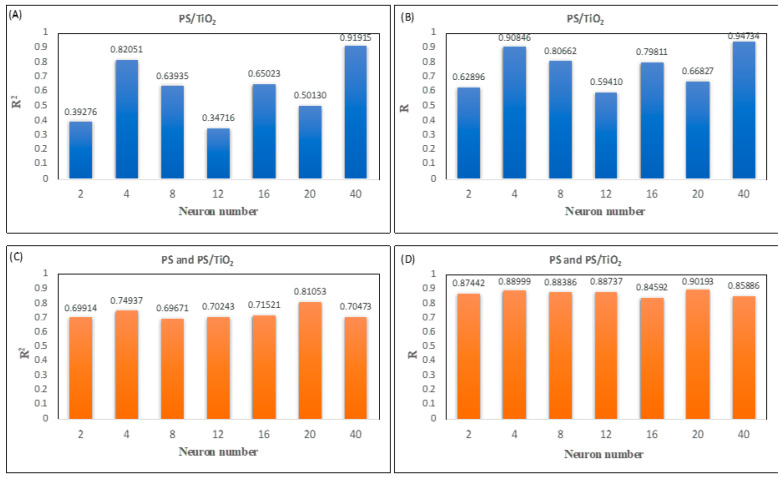
The results of designed MLP model for predicting diameters of (**A**,**B**) PS/TiO_2_ (Class 1) and (**C**,**D**) PS and PS/TiO_2_ (Class 2) nanofibers.

Figure 4A–D demonstrates R^2^ and R values obtained from MLP modeling of Class 1 and Class 2 nanofibers.

**Figure 5:**
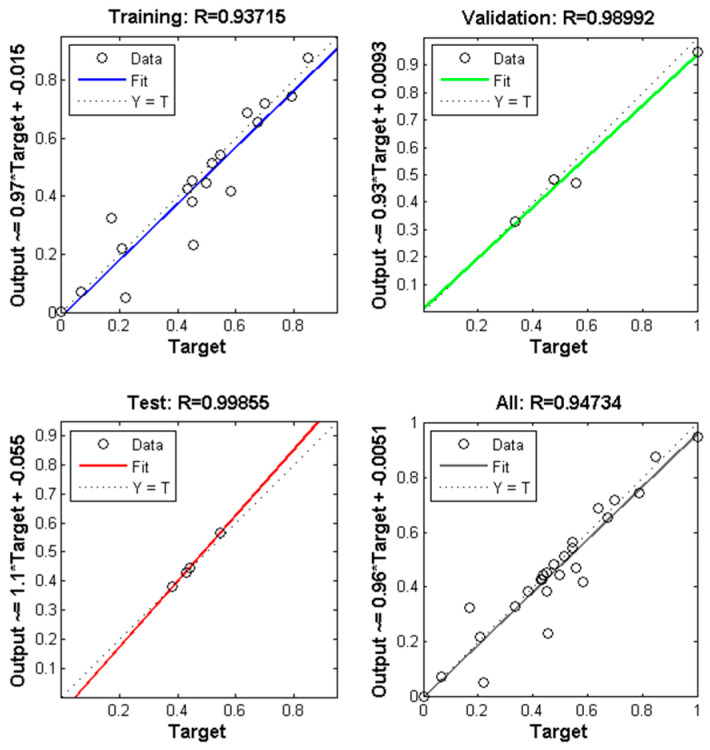
Plots of output versus target predicting the diameters of PS/TiO_2_ nanofibers by MLP network model.

**Figure 6:**
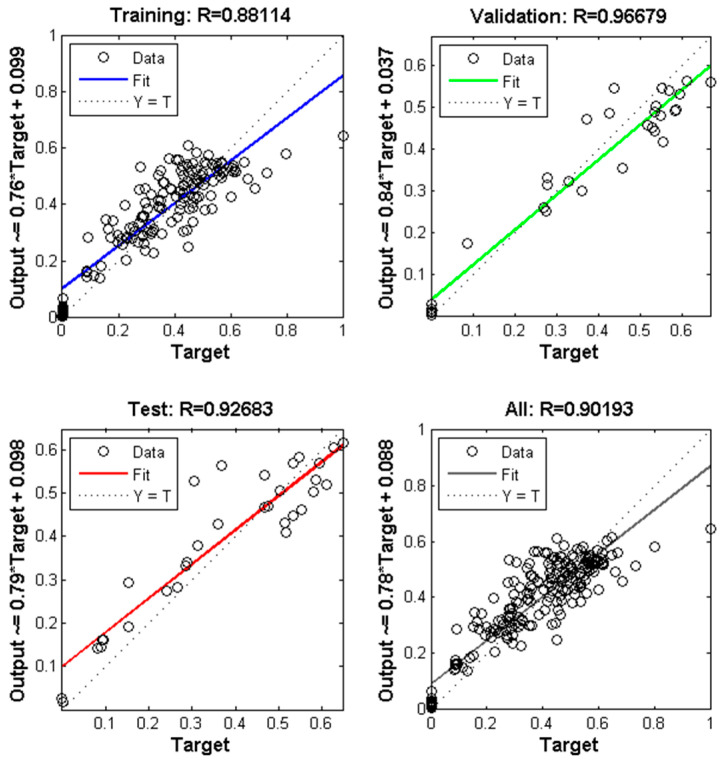
Plots of output versus target predicting the diameters of PS and PS/TiO_2_ nanofibers by MLP network model.

The results show that the highest R^2^ (0.91915) and R (0.94734) values were achieved for processing 40 neurons in the hidden layer for PS/TiO_2_ nanofibers (Figure 4A,B). For Class 2 nanofibers, processing 20 neurons in hidden layer outputs the highest R^2^ (0.81053) and R (0.90193) values (Figure 4C,D). The regression performance graphs for PS/TiO_2_ nanofiber and PS and PS/TiO_2_ nanofiber diameter prediction are presented in Figure 5 and Figure 6, respectively.

Scatter plots were used to assess the correlation between variables, thereby determining the accuracy of the MLP model. The regression coefficients (R) obtained from the comparison of MLP outputs and target values for PS/TiO_2_ nanofibers were 0.93715 (training), 0.98992 (validation), 0.99855 (testing), and 0.94734 (overall), as presented in Figure 5.

Similarly, for PS and PS/TiO_2_ nanofiber diameters, the corresponding regression values were 0.88114, 0.96679, 0.92683, and 0.90193 for training, validation, testing, and overall datasets, respectively (Figure 6). Based on the best prediction performance of nanofiber diameters, error analyses were conducted. The calculated values of MSE, MAE, and RMSE were 4.03 × 10^−3^, 5.80 × 10^−2^, and 6.35 × 10^−2^ for Class 1 and 7.01 × 10^−3^, 5.89 × 10^−2^, and 8.37 × 10^−2^ for Class 2, respectively.

#### 3.3.2. Validation Performance of MLP Model Outputs

**Figure 7:**
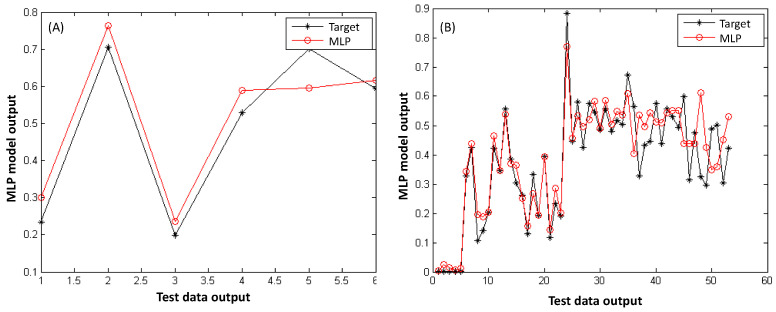
Target and simulated diameter of (**A**) PS/TiO_2_ nanofiber and (**B**) PS and PS/TiO_2_ nanofiber test data by MLP network model.

To verify the consistency of the test data with the MLP model calculations, the validation performance of the target and predicted outputs for all nanofiber classes is illustrated in Figure 7. As shown in Figure 7A, a clear and consistent agreement was observed between the target (experimental) and predicted values of PS/TiO_2_ nanofibers, demonstrating the reliability of the MLP network. Similarly, Figure 7B presents a strong agreement between the observed and predicted values for PS and PS/TiO_2_ nanofibers, further confirming the validation performance of the model.

### 3.4. RBF Network Modeling

The RBF neural network model was developed for all particle classes to achieve the maximum prediction accuracy, with the number of neurons varied and the spread constant set to 0.1, determined through trial-and-error optimization within the network structure. Previously, we demonstrated that in the model of the RBF network for prediction of the nanoparticle size, the error values dramatically decrease with the decrease in the number of spreads with the maximum number of neurons. Moreover, the best R and R^2^ values were achieved for the smallest spread numbers [38]. This finding is also confirmed in this study with the model of the RBF network for prediction of the PS nanofiber diameter. According to the results of training on 99 test data of PS nanofibers fabricated with DMF:THF solvent under varying spread constants (0.1–0.9) (Table 2), the minimum error was achieved with the spread number of 0.1. Thus, the following Class 1 and Class 2 nanofibers were designed with a spread number of 0.1 under varying numbers of neurons in the hidden layer.

#### Performances of Class 1 and Class 2 Nanofibers and Validation of RBF Model Outputs

**Figure 8:**
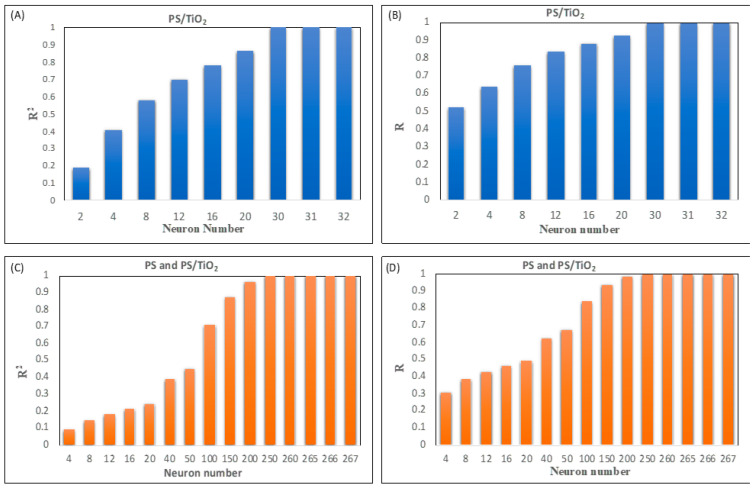
The results of designed RBF model for predicting diameters of (**A**,**B**) PS/TiO_2_ (Class 1) and (**C**,**D**) PS and PS/TiO_2_ (Class 2) nanofibers.

The highest determination coefficients (R^2^ and R) were obtained as the number of neurons approached the total number of data points for all nanofiber classes (Figure 8).

**Figure 9:**
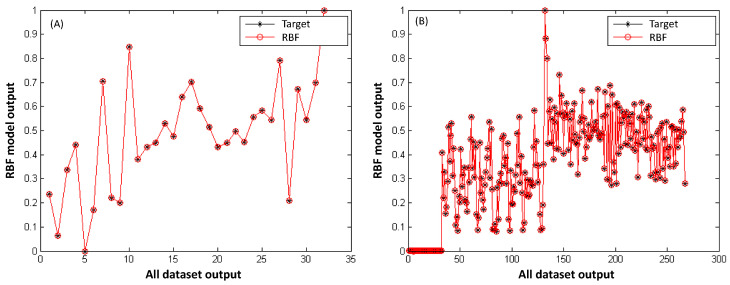
Target and simulated diameters of (**A**) PS/TiO_2_ nanofiber and (**B**) PS and PS/TiO_2_ nanofiber dataset by RBF network model.

Accordingly, the RBF model was constructed with 30 neurons for Class 1 and 250 neurons for Class 2 to predict nanofiber diameters. The agreement between experimental and predicted outputs further confirmed the validity of the RBF model, as illustrated in Figure 9. A strong consistency between observed and simulated values was observed for both classes (Figure 9A,B), demonstrating the reliability of the RBF approach.

### 3.5. ANN Models’ Comparison

Artificial neural networks (ANNs) encompass various architectures, among which MLP and RBF are the most widely applied for classification and regression tasks due to their ability to generalize from imprecise input data. While both exhibit a strong predictive performance, their learning mechanisms differ: RBF employs a localist learning approach that responds to specific regions of the input space, whereas MLP adopts a more distributed representation [47,57]. Previously, Yilmaz et al. reported a superior predictive performance of RBF compared to MLP in estimating soil swelling percentages [47]. In the present study, both MLP and RBF models were applied to predict nanofiber diameters obtained from SEM imaging. Comparative analysis revealed that the RBF model provided a higher accuracy and significantly lower error values than the MLP model (Table 3), underscoring its superior predictive capability for this application.

## 4. Discussion

The nanofiber diameter size plays a crucial role in determining their performance in various applications [58]. For instance, variations in the diameter of TiO_2_ nanofibers can strongly influence their functional properties, especially in applications related to optoelectronics and solar energy conversion [59]. However, precisely controlling the diameter of electrospun nanofibers is challenging due to the complex and time-consuming nature of the optimization of electrospinning parameters, which relies on trial-and-error approaches [60]. In this study, ANN-based prediction of the fiber diameter was developed, which can support the rational design and optimization of PS/TiO_2_ nanofiber membranes for filtration or antibacterial-related applications. The FTIR spectra confirm that TiO_2_ nanoparticles are successfully incorporated into the PS nanofiber matrix without altering the chemical structure of the polymer, ensuring stability of the composite membrane. The XRD patterns show that TiO_2_ retains its anatase crystalline phase after electrospinning, which is the most active phase for photocatalytic and antibacterial applications. Importantly, the observed inhibition of *E. coli* demonstrates that the embedded TiO_2_ remains functionally accessible at the fiber surface and is not deactivated by the polymer matrix. Together, these results confirm that the fabricated PS/TiO_2_ nanofibers are not only structurally well-formed but also functionally active.

Several machine learning algorithms have been proposed for nanofiber diameter prediction; among these, ANNs have been widely employed owing to their capacity to capture complex nonlinear relationships and their flexibility in modeling diverse types of data [61]. In ANNs, the computational model (neural structure) interconnects numerous units (processing elements), namely artificial neurons, through coefficients (weights) that encode the system’s memory [62]. This gives the advantage of employing the ANN method for the predictive analysis of nanofibers fabricated through the electrospinning process due to ANNs’ ability to accurately model complex systems with multiple input variables [63]. Several studies have investigated the prediction of nonlinear nanofiber diameter data derived from various polymers [7,10,22,23], and most of the reported studies employed feed-forward MLPs. In addition, previously reported results on several developed ANNs’ performances in terms of the predictive accuracy of nanofibers vary (R^2^: 0.99 for PAN [7], R^2^: 0.83 for gelatin [64], R^2^: 0.98 for PVP [24], R^2^: 0.97 for PCL [23], R^2^: 0.96 for PCL/gelatin [25], R^2^: 0.99 for PVA/chitosan/collagen [65]). Moreover, Lakshmi Narayana et al. developed an ANN model to predict the diameter of polycaprolactone (PCL) fibers, achieving R^2^ values of 0.97 for the training set and 0.98 for the test set [66]. Premasudha et al. proposed a more complex ANN-based model for predicting the diameters of polysaccharide (Hylon VII starch)-based biopolymer nanofibers, reporting a predictive accuracy of 95.2% [67].

Unlike single-layer perceptrons, which are limited to linear functions, MLPs with hidden layers can capture nonlinear relationships and are widely used due to the availability of diverse training algorithms [68]. Radial basis function (RBF) networks have also been introduced as alternatives to MLPs, offering faster training and improved convergence without entrapment in local minima [69]. In this study, given the nonlinear and complex nature of the input parameters related to process parameters of electrospinning, both MLP and RBF network models were developed, and their predictive performances according to nanofiber diameters were comparatively assessed. Class 1 and Class 2 of nanofibers are modeled through three and four input variables, respectively, with a one hidden layer of neurons. The hidden layer, located between the input and output layers of the network, consists of neurons that process and transmit information, thereby enhancing the accuracy of output parameter prediction [24,70]. Previously, Cuahuizo-Huitzil et al. reported the correlation coefficients as 0.96, 0.98, and 0.98 for ANN configurations with one, two, and three hidden layers, respectively [70]. Maurya et al. highlighted the significance of hidden layer neurons, testing configurations ranging from 2 to 15 neurons, and reported that a layer with 13 neurons yielded a minimum mean square error of 8.9 × 10^−5^ [71]. Given the sensitivity of predicting experimental nanofiber diameters, the optimal ANN structure was determined by varying the number of neurons in the hidden layer [8,16]. In this study, the number of neurons in each processing step dramatically affected the MLP prediction performances of Class 1 and Class 2 nanofibers (the best R = 0.94734 and R = 0.90193, respectively) as achieved through 40 neurons and 20 neurons in the hidden layer, respectively. The results indicated that increasing the number of neurons in the hidden layer did not necessarily improve the accuracy of output predictions for the experimental data. Similar results have also been reported by other authors [70]. An excessive increase in the number of neurons can lead to overfitting, limiting the model’s ability to generalize during the test phase and resulting in an overparameterized network [72]. The very low MSE values (in the order of 10^−32^) obtained for the RBF model reflect the interpolation nature of radial basis function networks when the number of hidden neurons approaches the number of training samples. Since training error alone does not reflect generalization and may raise concerns about overfitting, the model performance in this work was evaluated using k-fold cross-validation on unseen data. The best results of the performance of the RBF model (R = 1) were achieved with design parameters of 30 neurons and 250 neurons in the hidden layer of the model for the prediction of nanofiber diameters of Class 1 and Class 2, respectively. The obtained results revealed that if the neuron number is too small, the model cannot yield an accurate output value.

## 5. Conclusions

In this study, SEM images of PS/TiO_2_ and PS and PS/TiO_2_ nanofibers were analyzed using ImageJ to measure nanofiber diameters. The MLP and RBF models were employed for the nanofiber diameter prediction. The weighting of input and output variables for the two classes was optimized to determine the most effective network structure as a function of the number of neurons. The optimal ANN configuration was then selected based on the mean square error (MSE) and the correlation coefficient (R) between the predicted and experimental nanofiber diameters. The proposed models achieved successful predictions for all classes, with R values ranging from 0.90 to 1.0. The best prediction performance in terms of the MLP and RBF results was achieved with MSEs of 4.03 × 10^−3^ and 1.42 × 10^−32^ for Class 1, and 7.01 × 10^−3^ and 2.75 × 10^−32^ for Class 2, respectively. These results demonstrate the reliability and predictive power of both ANN approaches, particularly the RBF model. The findings suggest that similar artificial intelligence-based models could be effectively employed for predicting nanofiber diameters in future studies. Moreover, although this study focuses on the nanofiber diameter as the primary output variable, the developed ANN framework is inherently generalizable and can be extended to predict the porosity, pore size, and permeability by incorporating these parameters as additional outputs once the corresponding experimental datasets become available.

## Figures and Tables

**Table 1:** Simulation parameters.

Program	MATLAB R2016a	
	Class 1	Class 2
Number of neurons in input layer	3	4
Number of neurons in hidden layer	40	20
Number of neurons in output layer	1	1
Number of training datasets	26	214
Number of test datasets	6	53
Used ANN model	MLP	MLP
Epoch number in training process	1000	1000
Neuron number in training process	2–40	2–40
Type of learning algorithm	Levenberg–Marquardt algorithm	
Training activation function	Hidden Layer (TANSIG)	
	Output Layer (TANSIG)	
Used ANN model	RBF	RBF
Spread number in training process	0.1	0.1
Neuron number in training process	2–32	2–267
Initial learning rate	1 × 10^−7^	
Normalization	Min–Max Normalization	

**Table 2:** The values of the error parameters for tuning in RBF network model.

Spread Number	MAE	MSE	RMSE	R^2^
0.1	2.53 × 10^−16^	1.25 × 10^−31^	3.54 × 10^−16^	1
0.2	1.08 × 10^−15^	2.03 × 10^−30^	1.42 × 10^−15^	1
0.3	2.26 × 10^−13^	8.30 × 10^−26^	2.88 × 10^−13^	1
0.4	3.78 × 10^−11^	2.86 × 10^−21^	5.35 × 10^−11^	1
0.5	2.66 × 10^−9^	1.46 × 10^−17^	3.82 × 10^−9^	1
0.9	1.04 × 10^−14^	1.98 × 10^−8^	1.41 × 10^−4^	0.9999997

**Table 3:** Best performance indices (MSE, MAE, RMSE, R, and R^2^) for models.

MLP Model	MSE	MAE	RMSE	R	R^2^
PS/TiO_2_	4.03 × 10^−3^	5.80 × 10^−2^	6.35 × 10^−2^	0.94734	0.91915
PS and PS/TiO_2_	7.01 × 10^−3^	5.89 × 10^−2^	8.37 × 10^−2^	0.90193	0.81053
**RBF Model**	**MSE**	**MAE**	**RMSE**	**R**	**R^2^**
PS/TiO_2_	1.42 × 10^−32^	7.42 × 10^−17^	1.19 × 10^−16^	1	1
PS and PS/TiO_2_	2.75 × 10^−32^	1.02 × 10^−16^	1.66 × 10^−16^	1	1

## Data Availability

The original contributions presented in this study are included in the article. Further inquiries can be directed to the corresponding author.

## References

[1] Aydın R.S.T., Eroğlu A.N., Karakeçili A., Çalımlı A. (2016). Designing double-layered nanofibrous membranes as a wound dressing material. Fibers Polym..

[2] Gümüşderelioğlu M., Dalkıranoğlu S., Aydın R.S.T., Çakmak S. (2011). A novel dermal substitute based on biofunctionalized electrospun PCL nanofibrous matrix. J. Biomed. Mater. Res. Part A.

[3] Tamura T., Kawakami H. (2010). Aligned Electrospun Nanofiber Composite Membranes for Fuel Cell Electrolytes. Nano Lett..

[4] Kulkarni S.A., Mhaisalkar S.G., Mathews N., Boix P.P. (2019). Perovskite Nanoparticles: Synthesis, Properties, and Novel Applications in Photovoltaics and LEDs. Small Methods.

[5] Fang J., Wang X., Lin T. (2011). Functional applications of electrospun nanofibers. Nanofibers-Prod. Prop. Funct. Appl..

[6] Chen M., Patra P.K., Warner S.B., Bhowmick S. (2007). Role of fiber diameter in adhesion and proliferation of NIH 3T3 fibroblast on electrospun polycaprolactone scaffolds. Tissue Eng..

[7] Nasouri K., Bahrambeygi H., Rabbi A., Shoushtari A.M., Kaflou A. (2012). Modeling and optimization of electrospun PAN nanofiber diameter using response surface methodology and artificial neural networks. J. Appl. Polym. Sci..

[8] Brooks H., Tucker N. (2015). Electrospinning predictions using artificial neural networks. Polymer.

[9] Ahmadipourroudposht M., Fallahiarezoudar E., Yusof N.M., Idris A. (2015). Application of response surface methodology in optimization of electrospinning process to fabricate (ferrofluid/polyvinyl alcohol) magnetic nanofibers. Mater. Sci. Eng. C.

[10] Kalantary S., Jahani A., Pourbabaki R., Beigzadeh Z. (2019). Application of ANN modeling techniques in the prediction of the diameter of PCL/gelatin nanofibers in environmental and medical studies. RSC Adv..

[11] Zhou H., Shi Z., Wan X., Fang H., Yu D.-G., Chen X., Liu P. (2019). The relationships between process parameters and polymeric nanofibers fabricated using a modified coaxial electrospinning. Nanomaterials.

[12] Tao H.C., Wu T.Y., Aldeghi M., Wu T.C., Aspuru-Guzik A., Kumacheva E. (2021). Nanoparticle synthesis assisted by machine learning. Nat. Rev. Mater..

[13] Youshia J., Ali M.E., Lamprecht A. (2017). Artificial neural network based particle size prediction of polymeric nanoparticles. Eur. J. Pharm. Biopharm..

[14] Khan K.-U.-H., Siddiqui I.A. (2025). A predictive model for electrospun based Polyvinyl alcohol (PVA) nanofibers diameter using an artificial neural network. Sci. Rep..

[15] Ma M., Zhou H., Gao S., Li N., Guo W., Dai Z. (2023). Analysis and Prediction of Electrospun Nanofiber Diameter Based on Artificial Neural Network. Polymers.

[16] Sarkar K., Ghalia M.B., Wu Z., Bose S.C. (2009). A neural network model for the numerical prediction of the diameter of electro-spun polyethylene oxide nanofibers. J. Mater. Process. Technol..

[17] Khanlou H.M., Sadollah A., Ang B.C., Kim J.H., Talebian S., Ghadimi A. (2014). Prediction and optimization of electrospinning parameters for polymethyl methacrylate nanofiber fabrication using response surface methodology and artificial neural networks. Neural Comput. Appl..

[18] Nasouri K., Shoushtari A.M., Khamforoush M. (2013). Comparison between artificial neural network and response surface methodology in the prediction of the production rate of polyacrylonitrile electrospun nanofibers. Fibers Polym..

[19] Faridi-Majidi R., Ziyadi H., Naderi N., Amani A. (2012). Use of artificial neural networks to determine parameters controlling the nanofibers diameter in electrospinning of nylon-6, 6. J. Appl. Polym. Sci..

[20] Karimi M.A., Pourhakkak P., Adabi M., Firoozi S., Adabi M., Naghibzadeh M. (2015). Using an artificial neural network for the evaluation of the parameters controlling PVA/chitosan electrospun nanofibers diameter. e-Polymers.

[21] Rabbi A., Nasouri K., Bahrambeygi H., Shoushtari A.M., Babaei M.R. (2012). RSM and ANN approaches for modeling and optimizing of electrospun polyurethane nanofibers morphology. Fibers Polym..

[22] Ketabchi N., Naghibzadeh M., Adabi M., Esnaashari S.S., Faridi-Majidi R. (2017). Preparation and optimization of chitosan/polyethylene oxide nanofiber diameter using artificial neural networks. Neural Comput. Appl..

[23] Khatti T., Naderi-Manesh H., Kalantar S.M. (2019). Application of ANN and RSM techniques for modeling electrospinning process of polycaprolactone. Neural Comput. Appl..

[24] Nasouri K. (2018). Novel estimation of morphological behavior of electrospun nanofibers with artificial intelligence system (AIS). Polym. Test..

[25] Kalantary S., Jahani A., Jahani R. (2020). MLR and ANN approaches for prediction of synthetic/natural nanofibers diameter in the environmental and medical applications. Sci. Rep..

[26] Ma Z., Zheng Z., Zeng J., Feng G., Huang Y., Luo W., He M., Cui J., Liang Y.J. (2024). Artificial neural networks for the accurate prediction of the thickness of fiber membranes prepared by airflow-assisted electrostatic spinning. J. Appl. Polym. Sci..

[27] Rezaei B., Shoushtari A.M., Rabiee M., Uzun L., Turner A.P., Mak W.C. (2018). Multifactorial modeling and optimization of solution and electrospinning parameters to generate superfine polystyrene nanofibers. Adv. Polym. Technol..

[28] Uddin M.N., Desai F.J., Asmatulu E. (2020). Biomimetic electrospun nanocomposite fibers from recycled polystyrene foams exhibiting superhydrophobicity. Energy. Ecol. Environ..

[29] Wang L., Fu Q., Yu J., Liu L., Ding B. (2019). Nanoparticle-doped polystyrene/polyacrylonitrile nanofiber membrane with hierarchical structure as promising protein hydrophobic interaction chromatography media. Compos. Commun..

[30] Zulfi A., Munir M.M., Hapidin D.A., Rajak A., Edikresnha D., Iskandar F., Khairurrijal K. (2018). Air filtration media from electrospun waste high-impact polystyrene fiber membrane. Mater. Res. Express.

[31] Gaabour L.H. (2021). Effect of addition of TiO_2_ nanoparticles on structural and dielectric properties of polystyrene/polyvinyl chloride polymer blend. AIP Adv..

[32] Zhang X.D., Xiao G., Wang Y.Q., Zhao Y., Su H.J., Tan T.W. (2017). Preparation of chitosan-TiO composite film with efficient antimicrobial activities under visible light for food packaging applications. Carbohydr. Polym..

[33] Raut A.V., Yadav H.M., Gnanamani A., Pushpavanam S., Pawar S.H. (2016). Synthesis and characterization of chitosan-TiO:Cu nanocomposite and their enhanced antimicrobial activity with visible light. Colloids Surf. B.

[34] Wanjale S., Birajdar M., Jog J., Neppalli R., Causin V., Karger-Kocsis J., Lee J., Panzade P. (2016). Surface tailored PS/TiO_2_ composite nanofiber membrane for copper removal from water. J. Colloid. Interface Sci..

[35] Neppalli R., Causin V., Benetti E.M., Ray S.S., Esposito A., Wanjale S., Birajdar M., Saiter J.-M., Marigo A. (2014). Polystyrene/TiO_2_ composite electrospun fibers as fillers for poly(butylene succinate-co-adipate): Structure, morphology and properties. Eur. Polym. J..

[36] Madani M., Sharifi-Sanjani N., Hasan-Kaviar A., Choghazardi M., Faridi-Majidi R., Hamouda A.S. (2013). PS/TiO_2_ (polystyrene/titanium dioxide) composite nanofibers with higher surface-to-volume ratio prepared by electrospinning: Morphology and thermal properties. Polym. Eng. Sci..

[37] Threepopnatkul P., Kulsetthanchalee C. (2018). Effect of ZnO and TiO_2_ on properties of polystyrene/nitrile rubber electrospun fiber mats. Mater. Today Proc..

[38] Aydın R.S.T., Demir A. (2025). Artificial neural network prediction of TiO_2_-doped chitosan micro/nanoparticle size based on particle imaging measurements. Colloid Polym. Sci..

[39] Siddique M.A.B., Khan M.M.R., Arif R.B., Ashrafi Z. (2018). Study and observation of the variations of accuracies for handwritten digits recognition with various hidden layers and epochs using neural network algorithm. Proceedings of the 2018 4th International Conference on Electrical Engineering and Information & Communication Technology (iCEEiCT).

[40] Uzair M., Jamil N. (2020). Effects of hidden layers on the efficiency of neural networks. Proceedings of the 2020 IEEE 23rd International Multitopic Conference (INMIC).

[41] Lau E.T., Sun L., Yang Q. (2019). Modelling, prediction and classification of student academic performance using artificial neural networks. SN Appl. Sci..

[42] Adil M., Ullah R., Noor S., Gohar N. (2022). Effect of number of neurons and layers in an artificial neural network for generalized concrete mix design. Neural Comput. Appl..

[43] Fan L., An J., Gao J., Cui Y., Dong Z. (2022). Determination of atypical antipsychotics in human plasma by UPLC-UV with polystyrene nanofibers as a solid-phase extraction sorbent. RSC Adv..

[44] Uyar T., Besenbacher F. (2008). Electrospinning of uniform polystyrene fibers: The effect of solvent conductivity. Polymer.

[45] Pham B.T., Nguyen M.D., Bui K.-T.T., Prakash I., Chapi K., Bui D.T. (2019). A novel artificial intelligence approach based on Multi-layer Perceptron Neural Network and Biogeography-based Optimization for predicting coefficient of consolidation of soil. CATENA.

[46] Jahani A., Fazel A. (2016). Aesthetic Quality Modeling of Landscape in Urban Greenspace by Using Artificial Neural Network. J. Nat. Environ..

[47] Yilmaz I., Kaynar O. (2011). Multiple regression, ANN (RBF, MLP) and ANFIS models for prediction of swell potential of clayey soils. Expert. Syst. Appl..

[48] Foody G.M. (2004). Supervised image classification by MLP and RBF neural networks with and without an exhaustively defined set of classes. Int. J. Remote Sens..

[49] Sun B., Long Y.Z., Zhang H.D., Li M.M., Duvail J.L., Jiang X.Y., Yin H.L. (2014). Advances in three-dimensional nanofibrous macrostructures via electrospinning. Prog. Polym. Sci..

[50] Essalhi M., Khayet M., Cojocaru C., García-Payo M.C., Fernández P.A. (2013). Response Surface Modeling and Optimization of Electrospun Nanofiber Membranes. Open Nanosci. J..

[51] Eroğlu A.N., Aydin R.S.T., Karakeçili A., Çalimli A. (2017). Fabrication and Process Optimization of Poly(2-hydroxyethyl methacrylate) Nanofibers by Response Surface Methodology. J. Nanosci. Nanotechnol..

[52] Budlayan M.L., Patricio J., Oracion J.P., Capangpangan R., Arco S., Alguno A., Basilio A., Latayada F. (2021). Improvised centrifugal spinning for the production of polystyrene microfibers from waste expanded polystyrene foam and its potential application for oil adsorption. J. Eng. Appl. Sci..

[53] Shahshojaei M., Behniafar H., Shaabanzadeh M. (2013). Preparation and Characterization of Polystyrene/TiO_2_ Core-Shell Nanospheres via Suspension Technique. Adv. Mater. Res..

[54] Li W., Liang R., Hu A., Huang Z.-H., Zhou Y. (2014). Generation of oxygen vacancies in visible light activated one-dimensional iodine TiO_2_ photocatalysts. RSC Adv..

[55] Venugopal R., Kiran S., Sreenivas B. (2025). The Role of TiO_2_ Nanoparticles in Enhancing the Structural Properties and Thermal Stability of PVA Nanocomposites. Int. J. Sci. Res. Eng. Trends.

[56] Kadhum A. (2017). Antimicrobial Activity of TiO_2_ NPs against *Escherichia coli* ATCC 25922 and *Staphylococcus aureus* ATCC 25923. Int. J. Comput. Appl. Sci..

[57] McGarry K., Wermter S., Macintyre J. Knowledge Extraction from Local Function Networks. Proceedings of the International Joint Conference on Artificial Intelligence.

[58] Ding J., Zhang J., Li J., Li D., Xiao C., Xiao H., Yang H., Zhuang X., Chen X. (2019). Electrospun polymer biomaterials. Prog. Polym. Sci..

[59] Zatirostami A. (2021). Fabrication of dye-sensitized solar cells based on the composite TiO_2_ nanoparticles/ZnO nanorods: Investigating the role of photoanode porosity. Mater. Today Commun..

[60] Pham Q., Sharma U., Mikos A. (2006). Electrospinning of Polymeric Nanofibers for Tissue Engineering Applications: A Review. Tissue Eng..

[61] Sukpancharoen S., Wijakmatee T., Katongtung T., Ponhan K., Rattanachoung N., Khojitmate S. (2024). Data-driven prediction of electrospun nanofiber diameter using machine learning: A comprehensive study and web-based tool development. Results Eng..

[62] Agatonovic-Kustrin S., Beresford R. (2000). Basic concepts of artificial neural network (ANN) modeling and its application in pharmaceutical research. J. Pharm. Biomed. Anal..

[63] Abiodun O.I., Jantan A., Omolara A.E., Dada K.V., Mohamed N.A., Arshad H. (2018). State-of-the-art in artificial neural network applications: A survey. Heliyon.

[64] Naghibzadeh M., Adabi M. (2014). Evaluation of Effective Electrospinning Parameters Controlling Gelatin Nanofibers Diameter via Modelling Artificial Neural Networks. Fibers Polym..

[65] Pervez M.N., Yeo W.S., Mishu M.M.R., Talukder M.E., Roy H., Islam M.S., Zhao Y., Cai Y., Stylios G.K., Naddeo V. (2023). Electrospun nanofiber membrane diameter prediction using a combined response surface methodology and machine learning approach. Sci. Rep..

[66] Narayana P.L., Wang X.-S., Yeom J.-T., Maurya A.K., Bang W.-S., Srikanth O., Reddy M.H., Hong J.-K., Reddy N.G.S. (2021). Correlating the 3D melt electrospun polycaprolactone fiber diameter and process parameters using neural networks. J. Appl. Polym. Sci..

[67] Premasudha M., Reddy S.R.B., Yeon-Ju L., Panigrahi B., Cho K.K., Reddy N.S. (2020). Using artificial neural networks to model and interpret electrospun polysaccharide (Hylon VII starch) nanofiber diameter. J. Appl. Polym. Sci..

[68] Haykin S.S. (1999). Neural Networks: A Comprehensive Foundation.

[69] Park J., Sandberg I.W. (1991). Universal Approximation Using Radial-Basis-Function Networks. Neural Comput..

[70] Cuahuizo-Huitzil G., Olivares-Xometl O., Castro M.E., Arellanes-Lozada P., Meléndez-Bustamante F.J., Torres I.H.P., Santacruz-Vázquez C., Santacruz-Vázquez V. (2023). Artificial Neural Networks for Predicting the Diameter of Electrospun Nanofibers Synthesized from Solutions/Emulsions of Biopolymers and Oils. Materials.

[71] Maurya A.K., Narayana P.L., Bhavani A.G., Jae-Keun H., Yeom J.-T., Reddy N.S. (2020). Modeling the relationship between electrospinning process parameters and ferrofluid/polyvinyl alcohol magnetic nanofiber diameter by artificial neural networks. J. Electrost..

[72] Silva I., Spatti D., Flauzino R.A., Liboni L.B., Alves S.R. (2017). Artificial Neural Networks.

